# Measuring Heart Rate in Freely Moving Mice

**DOI:** 10.21769/BioProtoc.4926

**Published:** 2024-02-05

**Authors:** Jérémy Signoret-Genest, Nina Schukraft, Philip Tovote

**Affiliations:** 1 Institute of Clinical Neurobiology, University Hospital Wuerzburg, Wuerzburg, Germany; 2 Center for Mental Health, University Hospital Wuerzburg, Wuerzburg, Germany

**Keywords:** Heart rate, ECG, Mouse, States, Freely behaving

## Abstract

Measuring autonomic parameters like heart rate in behaving mice is not only a standard procedure in cardiovascular research but is applied in many other interdisciplinary research fields. With an electrocardiogram (ECG), the heart rate can be measured by deriving the electrical potential between subcutaneously implanted wires across the chest. This is an inexpensive and easy-to-implement technique and particularly suited for repeated recordings of up to eight weeks. This protocol describes a step-by-step guide for manufacturing the needed equipment, performing the surgical procedure of electrode implantation, and processing of acquired data, yielding accurate and reliable detection of heartbeats and calculation of heart rate (HR). We provide MATLAB graphical user interface (GUI)–based tools to extract and start processing the acquired data without a lot of coding knowledge. Finally, based on an example of a data set acquired in the context of defensive reactions, we discuss the potential and pitfalls in analyzing HR data.

Key features

• Next to surgical steps, the protocol provides a detailed description of manufacturing custom-made ECG connectors and a shielded, light-weight patch cable.

• Suitable for recordings in which signal quality is challenged by ambient noise or noise from other recording devices.

• Described for 2-channel differential recording but easily expandable to record from more channels.

• Includes a summary of potential analysis methods and a discussion on the interpretation of HR dynamics in the case study of fear states.

## Background

Over the past decades, measurements of cardiac function have been exploited not only in cardiovascular research but also in other disciplines like neuroscience due to their tight connection with neural processes and as readouts for emotional states (Carrive, 2000; Leman et al., 2003; Tovote et al., 2005; Stiedl et al., 2009). To take into account the integrated nature of cardiac and motor functions during emotional challenge, we recently developed a novel analytical framework. By analyzing cardiac parameters together with behavioral readouts, we unveiled critical cardio-behavioral states that would not have been detectable with either of the readouts alone (Signoret-Genest et al., 2023).

A variety of non-invasive and invasive methods have been established in order to measure cardiovascular parameters. Non-invasive approaches include surface recording (different electrodes are embedded in the floor and contacted by the individual paws) or external telemetry systems with instrumented jackets, which allow for simultaneous respiratory function monitoring (Chu et al., 2001; Sato, 2019; Fares et al., 2022). However, the signals acquired with these methods are prone to be noisy, compromising the final data quality, and the restricted experimental conditions (e.g., specific testing box) or added burden on the animal (jacket) might hinder the expression of naturalistic behaviors, thereby limiting their scope of application.

An electrocardiogram (ECG) records the electrical charge shifts that occur during a cardiac cycle. Non-tethered telemetry recording systems that record the ECG allow the animal to move freely and are thus ideally suited for long-term recordings (Calvet and Seebeck, 2023). However, they are both invasive, since they require a battery and components for wireless transmission, as well as rather expensive, and may additionally pose data synchronization challenges. On the other hand, tethered systems to record the ECG are inexpensive and easy to establish but must be optimized in order to successfully deal with potential hurdles such as environmental interferences or experimental artifacts.

Tethered ECG recordings, in particular, grant researchers direct access to raw data, which must then undergo proper pre-processing to extract the relevant biological phenomenon, namely heartbeats. As for any other technique, heart rate data then needs to be processed to answer biological questions. However, despite the apparent ubiquity of this readout, the selection of analyses, data sub-selection, treatment, and interpretation are not immune to pitfalls and must be approached with caution.

Here, we present a protocol for cost-effective, highly reliable, and user-friendly ECG recordings in freely behaving mice. The protocol encompasses the fabrication of the patch cable and ECG connector implants, detailed surgical implantation procedures, and subsequent data acquisition and processing steps. We also provide access to a custom graphical user interface (GUI) for heart rate extraction, along with specific (pre)processing strategies. We discuss data processing and interpretation through the scope of recent data, showing that there is more to heart rate–related readouts than simple averages or single heart rate variability (HRV) values, and that such minimalist processing could reduce the statistical power of studies or introduce biases.

## Materials and reagents

**Reagents**

1. Orthophosphoric acid (Carl Roth, catalog number: 6366.1)
2. Paladur, liquid component (Anton Gerl, catalog number: 82462)
3. Buprenorphin (Bayer, catalog number: 14439113)
4. Isoflurane (cp-pharma, catalog number: 1214)
5. Depilatory cream (Veet)
6. Cutasept F (Hartmann AG, catalog number: 9803650)
7. Naropin (Aspen Germany, catalog number: 02749854)
8. Vitagel (Bausch & Lomb, catalog number: 1318187)
9. Braunol (Braun, catalog number: 190971)
10. Metacam (Boehringer Ingelheim, catalog number: 08890217)

**Laboratory supplies**
1. Circular miniature connector, male (OMNETICS, catalog number: A79108-001)
2. Wire, stainless steel, 7 strand, PFA (AM Systems, catalog number: 793200)
3. Soldering tin (Stannol, catalog number: 574006)
4. Glue, transparent (Silisto, catalog number: 71024)
5. Ultra-flexible microminiature shielded cable (Daburn, catalog number: 2721/5)
6. Blunt needles, 27 G (Braun Petzold, catalog number: 9180117)
7. M9 connector, male (Binder, catalog number: 99-0413-00-05)
8. Heat shrink tubing set (Haupa, catalog number: 267200)
9. Regular insulated wire (RadioSpare, catalog number: 872-5167)
10. Circular miniature connector, female (OMNETICS, catalog number: A79109)
11. Teflon tape (Toolineo, catalog number: 100000000178797)
12. Aluminum foil
13. 1 mL syringes (Praxisdienst, catalog number: 138322)
14. Needles, 30 G (Praxisdienst, catalog number: 128330)
15. Cotton buds
16. Cotton wipes (Maicell, catalog number: 72100)
17. Glue, viscous (Pattex, catalog number: 2804443)
18. Scalpel blade (Hartenstein, catalog number: SJ10)
19. Suture material, ECG (Serag Wiessner, catalog number: LO07340B)
20. Suture material, skin, braided silk (SMI, catalog number: 8200518)
21. Toothpicks
22. Glue, black (Wekem, catalog number: WK-2400)

## Equipment

1. Third hand (Toolcraft, catalog number: TO-6871371)
2. Stereomicroscope (Olympus, model: SZ-61)
3. Forceps, straight (Fine Science Tools, catalog number: 11252-00)
4. Soldering station (Velleman, model: VTSSC40N)
5. Scissors (Passau Impex, catalog number: 14060-10)
6. Cable stripper (Toolcraft, catalog number: TO-4861971)
7. Lighter
8. Multimeter (Fluke, model: 179)
9. Balance (Kern, model: EMB 2200-0)
10. Anesthetizing box (Hugo Sachs Elektronik, catalog number: 50-0108)
11. Anesthesia mask (Hugo Sachs Elektronik, catalog number: 73-4858)
12. Anesthetic vaporizer (Hugo Sachs Elektronik, catalog number: 34-2052)
13. MiniVac gas evacuation unit (Hugo Sachs Elektronik, catalog number: 73-4910)
14. Fluosorber filter canister (Hugo Sachs Elektronik, catalog number: 34-0415)
15. Heating pad (Kent Scientific, model: RightTemp Jr.)
16. Forceps, curved (Fine Science Tools, catalog number: 11271-30)
17. Forceps, straight, blunt (Fine Science Tools, catalog number: 11002-14)
18. Feeding cannula (Fine Science Tools, catalog number: 18061-75)
19. Needle holder (Passau Impex, catalog number: T220218)
20. Scalpel handle (Fine Science Tools, catalog number: 10003-12)
21. Infrared lamp (Medisana, catalog number: 88232)
22. Amplifier with headstage (NPI Electronic, model: DPA-2FX)
23. Analog acquisition system with digitizer (e.g., Plexon Omniplex)
24. Large custom-made box (e.g., 1 m × 1 m × 1 m)
25. Custom-made Plexiglas cylinder (specifications depend on the conducted behavioral experiment; here, a cylinder with 30 cm diameter, 50 cm height, and a wall thickness of 0.4 cm was used)

## Software and datasets

**Acquisition software**

Dedicated software associated with a system to record analog signals. The data presented here was recorded with a digital Omniplex system (Plexon, Dallas, TX, USA) designed for electrical recordings of neuronal activity.

**Analysis software**

1. MATLAB 2022a (back compatibility at least until release 2019a; MathWorks, 09/03/2022)
2. ECG_Process package (custom-written code); current version can be found on: https://github.com/Defense-Circuits-Lab/ECGanalysis
3. Prism v9.5 (GraphPad, 26/01/2023)

## Procedure

- **Manufacturing of ECG connectors**
*See General Note 1.*
  1. The circular miniature connector has six pins, from which three are used for manufacturing the ECG connector. Fix the miniature connector in the clamp of a third hand and bend all five peripheral pins to 90°. Pinch the central pin with fine forceps and twist it to break it close to the base (Figure 1).
*Note: If there is no use for the additional pins, they can also be removed or bent on the sides to increase support.*
    **Figure 1.**
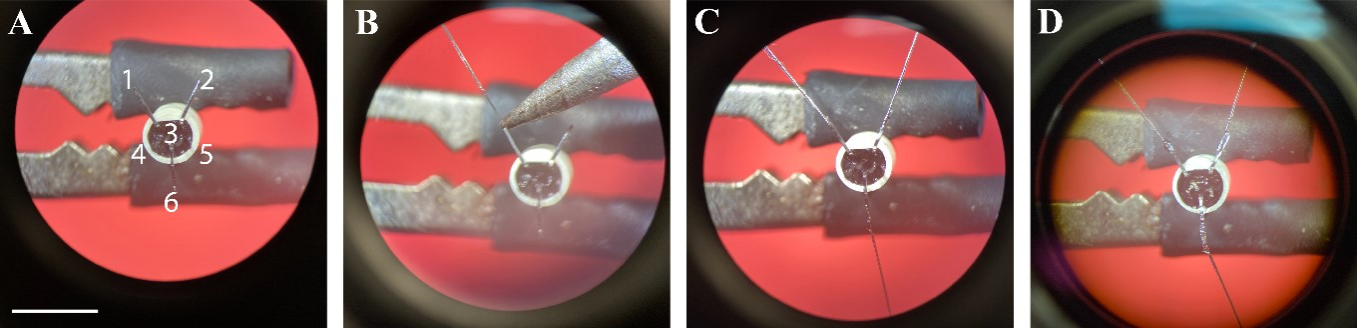
Manufacturing of electrocardiogram (ECG) connectors. A. Three pins are bent towards the outside and the three remaining pins are cut short. B. The stripped end of the wire is soldered onto the pin. C. A second ECG and a grounding wire are soldered to the two other pins. D. A layer of glue is applied to the inside of the connector and on all pins and stripped wire parts. Scale bar: 5 mm.
  2. Solder two wires onto the miniature connector pins #1 and #2:
    - Cut two pieces of wire to a length of 7 cm.
    - Strip 5 mm from one side of the wire with fine forceps.
    - Add a very small amount of orthophosphoric acid on the pin.
    - Align the stripped wire onto the pin.
    - Take a small amount of soldering tin on the iron and briefly touch the pin/wire ensemble.
*Note: Soldering should feel easy—the melted tin should instantly run onto the pin, making a distinctive sound. The result should be a very thin soldering, looking shiny. The tin should not be kept on the iron for too long beforehand, and the iron should not remain on the pin beyond an instant, as it could loosen it internally by heating the connector.*
    - Repeat the steps for pin #2.
  3. Solder a reference wire with a length of 2 cm onto connector pin #6 by repeating step A2.
  4. Test the soldering quality; pulling each wire with a quick and firm motion allows to assess whether the wires are properly soldered onto the pins.
*Note: A good soldering can withstand substantial pulling, which is a predictor for high-quality recordings.*
  5. Apply glue to all connections and stripped pieces of wire.
  6. Check the connections, fitting a dedicated female connector (same model as for the cable) onto the connector. Strip the very tip of the electrodes’ wires. Contact each pair of female connector’s pin/electrode with fine tools mounted on a multimeter to check for proper connection. Just in case, while still touching each wire, touch the successive pins on the female connector; only the proper one should have a low resistance.
- **Manufacturing the patch cable**
*See General Note 2.*
  1. Cut the appropriate length of microminiature shielded cable. This should allow the cable to run freely from the headstage to the mouse’s head while being held by a pulley, taking into account possible loops that appear during long recordings.
*Note: Potential damage is usually located on the mouse’s side and is due to bites or twisting-induced rupture of a wire inside the insulation. To save time and resources, it makes sense to make the cable much longer so that only the mouse’s side can be remade when necessary. The ideal length depends on how the extra cable length can be handled without risking damage through bending or rolling.*
  2. Assemble the headstage side of the cable (Figure 2).
*Note: It is advisable to start on this side to be able to check that the sheath properly connects the distal end of the mouse side, shielding all the way to the wire on the headstage side.*
    **Figure 2.**
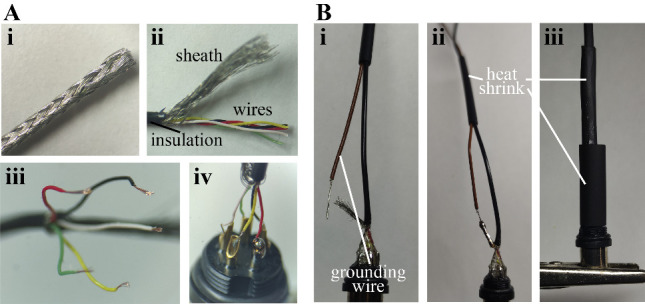
Manufacturing of the patch cable’s headstage side. A. Preparation of the miniature shielded cable. The cable is deinsulated (i) and the sheath is carefully unbraided (ii). The individual wires are unwinded, deinsulated (iii), and soldered onto the pins of the connector (iv). B. An extra wire is used as the grounding reference (i) by soldering it onto the sheath of the patch cable (ii). Heat shrink tubes of different sizes are used to insulate the connection (iii).
    - Begin by sliding the M9 connector casing onto the cable, followed by heat shrink sleeves of varying diameters: one nearly as large as the threaded part of the connector, one that fits the cable loosely, and one of intermediate size.
    - Carefully remove the insulation over approximately 1 cm and gently unbraid the sheath by using the tip of a blunt needle to wedge between the weave and pull towards the cut side. Loosely bundle the sheath on one side. Unwind the individual wires and arrange them according to Figure 2.
*Note: Avoid applying excessive force, as the insulation on the individual wires is delicate and inadvertent damage could lead to discarding the final product due to internal short circuits.*
    - Strip the end of the wires over 1–2 mm.
    - Maintain tight strand alignment, briefly dipping them into a drop of orthophosphoric acid placed on a surface, and then apply soldering tin.
*Note: Ensure that no single strand from any of the individual wires protrudes, as it may complicate the subsequent steps. Additionally, be aware that the wires’ insulation tends to shrink significantly when exposed to excessive heat, so exercise caution during soldering.*
    - Position the cables so that the end to be soldered is placed on top and aligns with the pins of the connector. You can achieve this by securing the cable around one of the binocular knobs and rotating the connector using a third hand tool, positioning the pins in front of the corresponding wires.
    - Apply a very small drop of acid to each pin.
*Note: Use only a minimal amount of acid, as excessive use may lead to excessive corrosion over time.*
    - Insert the first wire into the first pin and apply some soldering tin. The solder should be heated until it becomes fully liquid on the pin but take care not to overheat it, as it could melt the insulation on the thin wire. Repeat this process for the remaining pins.
    - Adjust the angle of the connector to ensure perfect alignment with the cable. Apply tension to the cable, for example by gently pulling it and adding a weight to prevent it from becoming slack.
    - Apply glue to the pins and wires, starting from the bottom of the pins and progressing to the top of the stripped part of the cable. Do this in several stages, incrementally adding glue and using Paladur's solvent to quickly cure the adhesive.
*Note: Be mindful not to extend the glue beyond the normal dimensions of the connector. Pay attention to the fact that the glue may briefly become more liquid after the application of Paladur's solvent.*
      **Critical:** Starting from this point, the connection between the non-deinsulated and insulated parts of the cable becomes extremely fragile, and the wires can easily break. Avoid bending or twisting them at all.
    - Move the smallest piece of heat shrink tubing closer to the connector.
    - For the grounding wire, prepare a piece of regular wire by deinsulating ~1 cm and coating the exposed wires with soldering tin.
    - Insert this wire into the piece of heat shrink tubing (along with the cable that is already inside) and position the exposed wire next to the sheath.
    - Apply a small amount of acid and solder both components together.
    - Gently slide the smallest heat shrink tubing all the way down to the glued section and then apply heat.
      **Critical:** Be cautious not to apply too much heat or for too long when shrinking the tubing. The insulation of the individual wires within the cable is very thin and can easily melt.
    - Successively add and shrink the other two pieces of tubing.
      **Critical:** Aim to keep the assembly as straight as possible. Otherwise, it might make it difficult to put the connector's casing in place.
    - Carefully slide down the various components of the connector. The cable gland contains *teeth* that will be tightened by the distal nut to grip the cable, so it is advisable to disassemble the connector into its different parts.
      i. Start with the main shaft, which should be screwed onto the part of the connector where the pins were.
      **Critical:** When screwing, ensure that the shaft does not twist the heat shrink and, consequently, the entire cable/wire assembly. Since they are soldered together, twisting could cause them to snap.
      ii. Then, add the *teeth*.
      iii. Finally, screw the nut onto the main shaft to secure and tighten the teeth, ensuring a firm grip on the heat shrink section.
  3. Fabricate the mouse side of the cable (Figure 3).
    **Figure 3.**
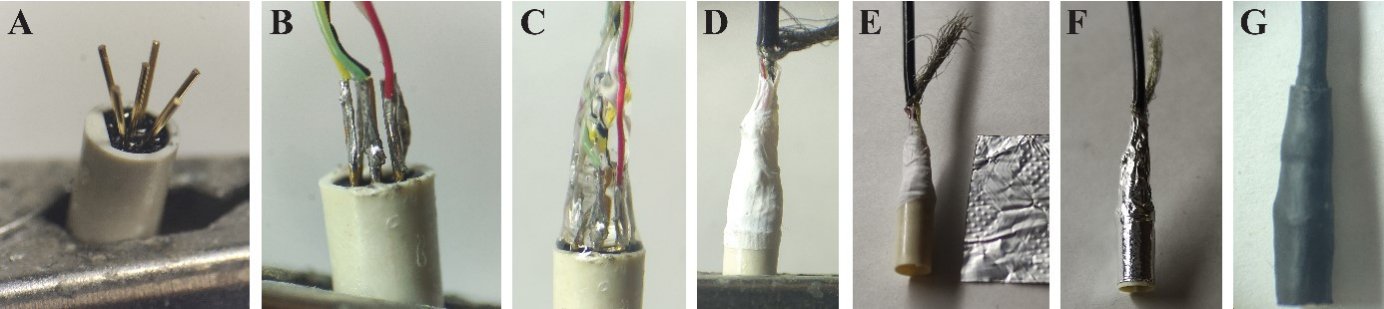
Manufacturing of the patch cable’s mouse side. A. Bent pins of the miniature connector held in a crocodile clamp. B. Thin soldered connection between individual wires and pins of the connector. C. Embedding in glue. D. Two layers of Teflon tape are wrapped around the connection. E. A piece of aluminum foil is prepared to insulate the connection. F. Wrapped aluminum foil. G. Two heat shrink tubings build the cover of the connection.
    - Begin by sliding two heat shrink pieces of different diameters: one slightly larger than the connector and the other with a loose fit around the cable.
*Note: If you want to exercise extra caution or if you have doubts about the headstage side, now is an opportune moment to check if the pins on the connectors at the headstage side are still properly connected to the individual wires by testing for a continuous electrical connection and no between-wires short circuits. Use a multimeter in resistance checking mode for this purpose.*
    - Prepare the cable end as previously instructed but strip less of the insulation.
    - Secure the connector in a crocodile clamp, bend the pins of the connector outward, and position the cable so that it lays on top of them.
    - Solder the individual wires according to the provided figure.
      **Critical:** The soldering should be as thin as possible and exhibit a shiny appearance.
    - Return the connector pins to a straight position.
*Note: Ensure there are no short circuits (solder touching another solder or a neighboring pin). If necessary, the pins can be bent laterally as long as the external diameter remains within the connector's limits.*
    - Embed the assembly in glue, trying to bring the wires closer together as they approach the cable.
    - Cut a piece of Teflon tape and wrap it around the glue, without deforming it first. Apply at least two layers. This step is crucial to ensure proper insulation between the soldering and the shielding in case the glue does not cover the entire area.
*Note: To prevent the Teflon from shifting, it is advisable to add a thin film of superglue to the already cured glue just before placing the Teflon.*
    - Prepare a piece of aluminum foil that matches the distance between the bottom of the connector and the stripped part of the cable. Apply glue to the connector and up to the cable (avoiding the sheath), and securely affix one layer of aluminum foil. Trim any excess foil.
    - Spread the cable sheath over the foil layer. Lower the tightest piece of tubing and shrink it.
    - Check the reference wire by confirming that the wires’ headstage side is well connected to the aluminum foil layer by using a multimeter.
    - Carefully lower the other piece until it reaches the edge of the connector (but avoid excessive extension) and then shrink it.
    - Ensure that all wires are correctly connected. Secure both connectors in clamps and use a multimeter with a fine tool (or a wire) to verify that each pin is linked exclusively to its corresponding counterpart and no other (Figure 4 ).
    **Figure 4.**
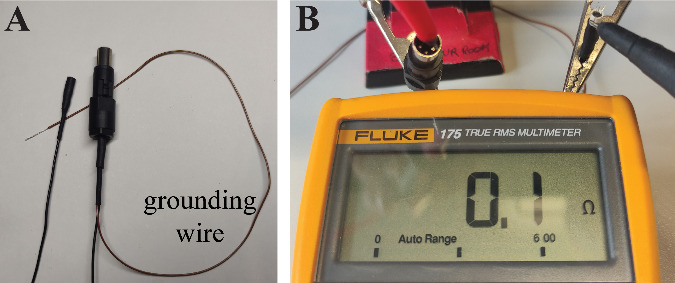
Final check of proper wire connection. A. Both ends of the cable are completed. B. A multimeter is used to confirm that all pins of the headstage side are properly connected to the cable’s mouse side connector.
- **Surgery: Implantation**
  1. Prepare the surgical field and all equipment needed throughout the surgery (Figure 5).
    **Figure 5.**
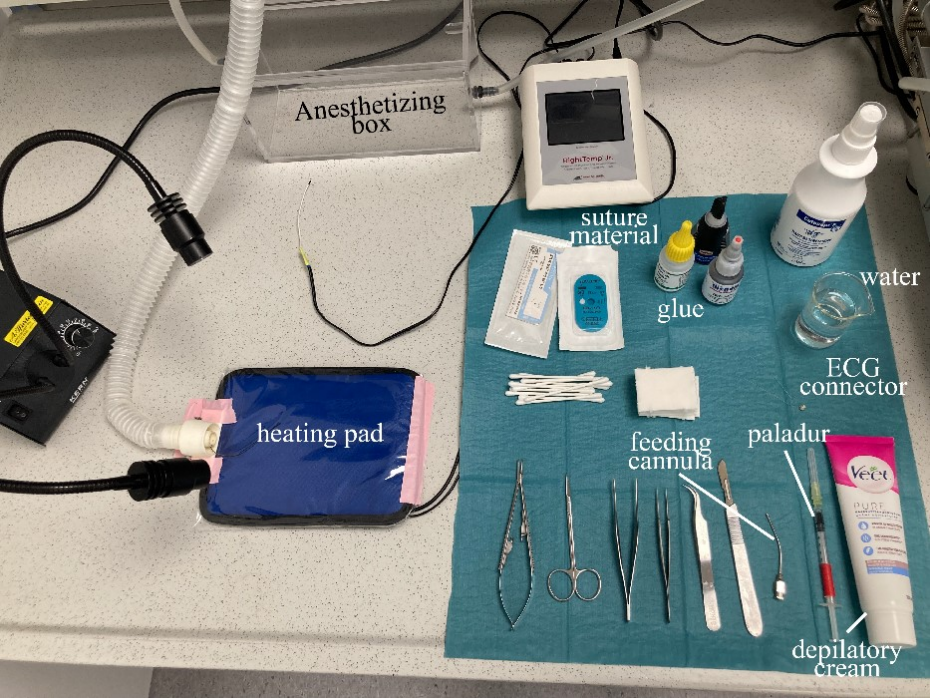
Preparation of the surgical field
  2. Prepare the animal (Figure 6A).
    **Figure 6.**
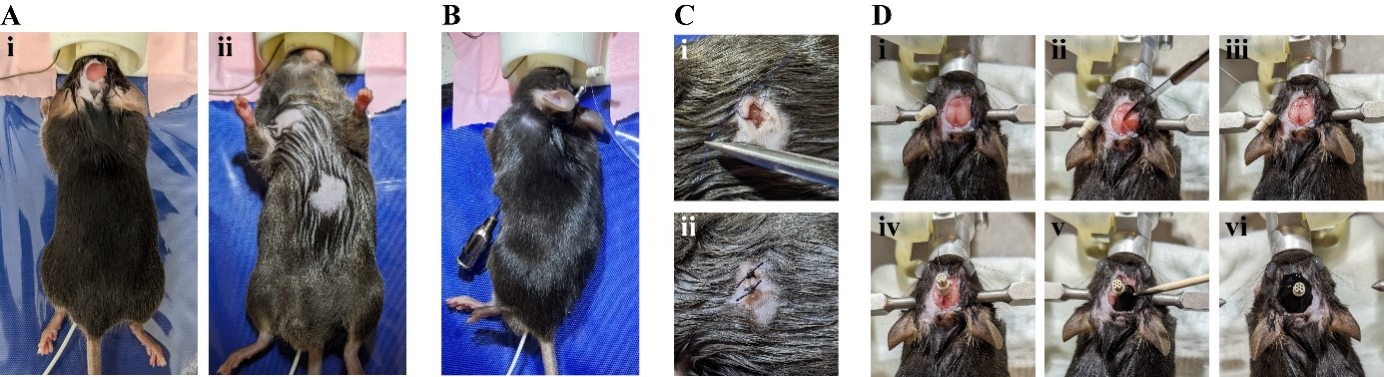
Surgical procedure of electrocardiogram (ECG) connector implantation. A. Dorsal (i) and ventral (ii) view of the mouse after preparing it for the implantation of the wires. B. The feeding cannula is inserted subcutaneously from the chest towards the incision on the head. The cable of the ECG connector is threaded through the hollow cannula. C. A small ball of glue is applied to the end of the stripped wire and a knot to suture the wire on the underlying muscle tissue is placed closely to it (i). The incision on the skin is sutured with 2–3 knots (ii). D. Exposed skull surface is dried (i) and roughened (ii, iii). The connector is glued onto the skull (iv) and a layer of black glue is added (v, vi).
    - Weigh the animal and inject the adequate amount of Buprenorphin (dosage 0.1 mg/kg) subcutaneously in the neck area 20–30 min prior to the start of the surgery for perioperative analgesia.
    - Anesthetize the animal by placing it into the isoflurane induction chamber (4% induction, 0.6 L/min oxygen flow).
    - Transfer the mouse on the heating pad and place the head in the anesthesia mask.
    - Check for paw reflexes to ensure deep anesthesia has been reached.
    - Maintain anesthesia with 1.5% isoflurane in oxygen.
    - Apply depilatory cream on top of the head to remove hair from a 1 × 1 cm area.
    - Turn the animal on its back and apply depilatory cream on the upper right and lower left of the animal’s chest.
    - Bring it back into prone position to access the head again.
    - Disinfect the skin by applying Cutasept three times and place 200 µL of Naropin using a 1 mL syringe directly underneath the skin on the top of the skull for local anesthesia.
  3. Open the skin.
    - With sharp scissors, cut off a 0.5 × 0.5 cm patch of skin on the head.
    - Turn the animal into supine position and disinfect the skin on the chest by applying Cutasept three times.
    - Cut a small incision into the skin on the chest with sharp scissors (3–5 mm).
  4. Thread the ECG wire (Figure 6B).
    - Use blunt forceps to carefully dissect the underlying tissue from the skin.
    - Insert the feeding cannula into the incision and gently push it dorsally towards the opened skin on the head.
      **Critical:** Always keep the cannula’s angle such that the tip faces outwards to prevent damage to any organs. Sliding the cannula should not require a lot of strength. If it feels otherwise, it is likely that the cannula is not being threaded subcutaneously. Keep the cannula in sterile water before insertion for it to glide smoothly onto the tissues and sterilize it thoroughly afterwards.
    - Pass the proper wire from the ECG connector through the hollow feeding needle from the head side. When the wire reaches the chest opening of the cannula, carefully pull the cannula out, leaving the wire in place.
*Note: Some conjunctive tissue can obstruct the cannula. In that case, just poke it with a needle to free the hole. Once the cannula is out, remove all tissue and sterilize it. Try to consistently use the wire that will be on the right side of the connector for the right chest, and similarly for the left.*
  5. Prepare the wire.
    - Strip 5 mm of the wire tip by using fine forceps.
    - Apply viscous glue to the very end of the wire tip. Do not spread it too much and make sure you leave enough stripped wire. Polymerize the glue by applying a small amount of Paladur’s solvent.
*Note: The tip of the wire can be pressed between some forceps to flatten it (just the tip) so that the individual strands are slightly spread, making it easier to keep glue there.*
  6. Fix the wire and skin suture (Figure 6C).
    - Dissect conjunctive tissues with forceps to expose the muscle and suture the wire onto it, going perpendicular to the muscle fibers. The knot should be placed close to the glue.
*Note: Leave the wire long enough that it does not impede the animal’s locomotion afterwards. It is possible and better to keep a small loop on the head side, which is then fitted subcutaneously.*
    - Close the skin with two interrupted sutures and disinfect the wounds with iodine-based disinfectant (e.g., Braunol).
  7. Repeat steps C4–C6 on the upper right of the chest with the second wire of the ECG connector.
  8. Fix the connector on the mouse’s head (Figure 6D).
    - Turn the animal into prone position again. Remove any remaining tissue from the skull bone by rubbing it with a cotton bud. Carefully roughen the skull surface by slightly scratching it with the back of a scalpel blade.
*Note: Proper fixation of the head in a stereotactic frame facilitates these steps, but this can also be performed in the anesthesia mask.*
    - Glue the miniature connector onto the skull and polymerize the glue with a small amount of Paladur’s solvent.
*Note: The skull needs to be absolutely dry.*
    - Strip a small portion of the remaining wire and slide it subcutaneously before gluing it into place by applying glue on the bone.
    - Form a small head cap by adding more glue around the connector and polymerize it.
    - Apply a layer of opaque black glue.
      **Critical:** Make sure that enough skull surface is exposed and recruited for the cap.
  9. Inject metacam (dosage 2 mg/kg) subcutaneously, transfer the animal to its cage, and let it recover under an infrared lamp.
  10. Monitor the animal until it has fully recovered from the anesthesia.
  11. Monitor the animal’s recovery every 12 h for 7 days post surgery.
  12. For long-term post-surgery care, monitor weight and general state daily.
*Note: Pay close attention to the sutures. This surgery is usually very well tolerated and animals fully recover behaviorally after approximately 1 h and regain their pre-surgery weight after 3–4 days.*
*See General Note 2.*
  13. (Optional) At the end of the experiments, retrieve the head cap and place in acetone overnight. Acetone will dissolve the glue but will not damage the connectors if left only for half a day. Rinse with water and dry. Check carefully for pin integrity and general condition. The connector can be reused a few times if handled with care.
    **Critical:** Acetone will dissolve and dilute the cyanoacrylate glue but upon evaporation it could still leave a layer of glue; several head caps can be placed in acetone at the same time but not too many, and the amount of acetone should be consequent. If left too long in acetone, the connectors will also start being damaged; check periodically whether the glue is dissolved and change the acetone solution if needed. Take extra care when checking the connectors before reusing them: connect a female connector and make sure that there is a connection between each pin of the female connector and its corresponding pin on the (reused) male connector. Also check each “ready-to-implant” connectors as described in step A6.
- **Data acquisition**
  1. Let the animal recover for at least one week before data acquisition.
  2. Familiarize the animal with handling and the connection procedure over several days.
*Note: Handling the animal for a minimum of three days significantly improves subsequent connection procedures. On the first day, allow the animal to explore the palm of the hand multiple times. On subsequent days, gradually habituate the animal to being held in the hand by gently gripping the head cap with the index finger and thumb while securing the tail with other fingers to prevent escape.*
  3. The day of the recording, connect the animal to the patch cable and place it in a behavioral arena, ideally with constant video monitoring.
*Notes:*
    - *A commutator could be used to prevent entanglement, but a simple pulley system with two grooved wheels, a lightweight thread, and a 5 mL syringe as a counterweight works well. Gently tie the thread to the cable and the syringe head. Adjust the counter pull by modifying the water level in the syringe. The ideal knot position on the cable should be determined empirically (Figure 7)* .
    - *Try as much as possible to only turn the amplifier on whenever the cable is connected to a mouse to prevent saturation of the system and potential damage.*
      **Figure 7.**
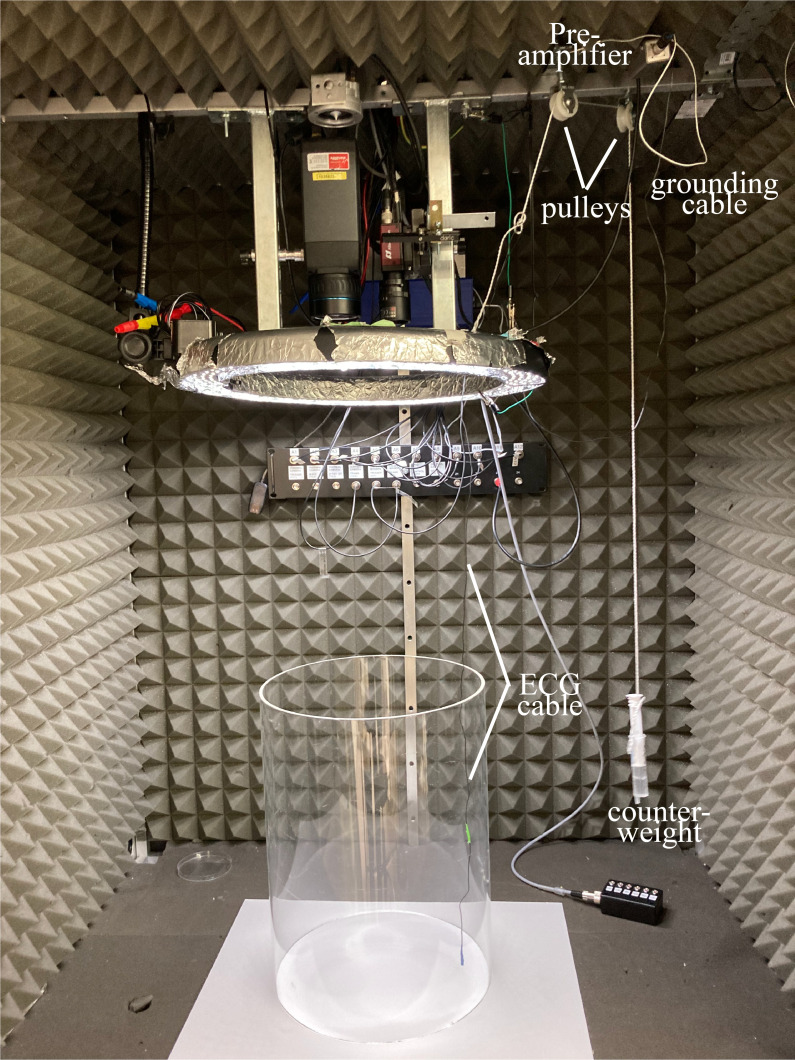
Recording setup with pulley system. A thread is tied to the electrocardiogram (ECG) cable and runs around two pulleys. An adjustable counterweight is tied to the other end of the thread.
  4. Turn on the acquisition system that receives the amplifier’s output to display it online and save it to a file. In order to preserve enough resolution in the ECG signal and allow for accurate heartbeat timestamping, the sampling rate should ideally be 5 kHz or higher (keep it mind that too high is not useful and will increase file size and processing time).
*Note: The current example is based on an NPI amplifier and a Plexon acquisition system (Omniplex/Cineplex).*
*Note: For file format considerations and pre-processing, see Data analysis section A.*
    **Critical:** If experiments involve aligning ECG data with behavioral data, it is essential to ensure perfect synchronization between the ECG signal and the video recording system. This synchronization should be carefully planned before the experiments begin. Recording systems rely on internal *clocks* to sample signals, and while these clocks are generally quite accurate individually, issues can arise when dealing with two separate systems and their clocks:
    a) They may start at different times (one system might be slower to start or require manual triggering), leading to an initial time lag (shift).
    b) Their clocks may run slightly faster or slower than each other, resulting in time discrepancies. For example, what appears as t = 10 min on one system could be t = 9 min 56 s on the other (drift).
    Addressing these synchronization challenges upfront is crucial for precise data alignment and to prevent any misinterpretation further down the line.
  5. Adjust the amplifier settings (these settings may vary between systems, but the following parameters are commonly present):
    - **Channels:** There is a reference channel and one “signal” channel for each electrode. In any case, the signal for each electrode is the voltage difference between the reference and that electrode. However, for each channel, we can also choose to output the difference between the two electrodes.
*Note: Unless one electrode's signal is significantly bad (contaminated with noise), it is a good idea to set one channel to output the differential signal and keep the other as a single output, using the better channel for this purpose. This choice should be made individually for each mouse, which should not lead to analytical issues, except for very specific analyses (e.g., interested in heartbeat morphology) where the configuration would matter.*
    - **Filters:** The low-pass and a high-pass filters can be adjusted. The low-pass filter keeps lower frequencies in the signal while reducing higher frequencies. Conversely, the high-pass filter removes low frequencies and keeps high frequencies.
      i. Low-pass filter: Somewhere around 1 kHz.
      ii. High-pass filter: A value is set to remove the slow fluctuations in the ECG signal. 30 Hz is a good option. If the amplifier features a 50 Hz (or 60 Hz, depending on the local power grid) notch filter, it can be beneficial to activate it when the signal is affected by prevalent grid-related interferences.
*Note: The amplifier’s analog filters do not sharply cut off specific frequencies in the signal. Instead, they have a gradual effect on the frequency spectrum. For example, if a high-pass filter is set at 30 Hz, it will reduce lower frequencies while allowing higher frequencies to pass through to varying degrees. It can be thought of as a gentle slope in how it affects the different frequencies. This means that when setting these filters, it is essential to consider the scope of the study. If the goal is not to study heartbeat morphology, it seems reasonable and more robust to directly adjust the filter settings to improve heartbeat separability from background over wave structure.*
    - **Gain:** Ensure that the gain is set high enough to clearly visualize the signal without saturating it, which means avoiding the signal hitting the upper or lower boundaries of the acquisition system. If saturation occurs, the signal will appear as a flat line at those extreme values.
*Note: Refer to “Troubleshooting Problem 2” for a comprehensive guide and tips on initially setting up the equipment and identifying potential issues.*
    - **Offset:** The offset allows you to adjust the baseline level of the signal, effectively moving it up or down. Aim to position the baseline (typically found between heartbeats) close to the middle values of the acquisition system, e.g., approximately 0 V if the system’s range goes from -5V to 5V. This adjustment helps to maintain the signal within the system’s optimal operating range.

## Data analysis

- **Heartbeat extraction**
  In this section, the process of extracting heartbeats using custom MATLAB code is described. This extraction is user friendly and does not require any coding experience. We are providing MATLAB code as regular and live scripts to allow to perform the different steps from the associated data set.
  1. Loading the data.
    The GUI accommodates various data sources, including simple times/values series in text/.csv files, .mat files, .pl2 files (Plexon), and .tsq files (Tucker-Davis).
*Note: While most systems allow to export data as csv files, the list of supported file formats can easily be expanded as needed. It only requires either the dedicated API for MATLAB to access the proprietary file format or, in other cases where the files are disguised text files, knowledge about the headers and data organization within the file.*
    - Click *Start path* to select a starting folder (optional, for convenience).
    - Click *Load file* to choose a data file. You can also select "_HeartBeats.mat" files (output files) to reload the analyses within the GUI (Figure 8).
      **Figure 8.**
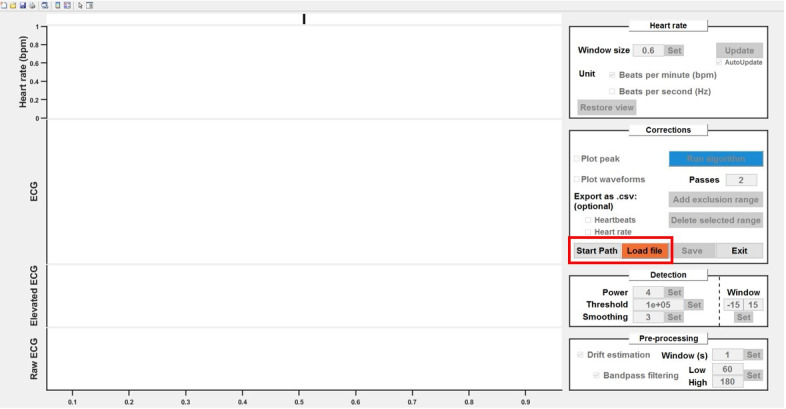
Load files into the processing graphical user interface (GUI). As an option, you can define a “start path” to select batch folders. Click *load file* to choose the first file to be pre-processed.
  2. Raw ECG pre-processing.
    Raw ECG signals can undergo pre-processing, which includes:
    - Detrending: Originally designed for human recordings with significant baseline shifts, this feature could also benefit recordings from other systems. It involves computing a sliding mean with a large window (covering multiple heartbeats) and subtracting it from the raw signal.
    - Bandpass filtering: A zero-phase bandpass filter can be applied to remove slow fluctuations and noise (zero-phase filtering ensuring no signal shift).
      i. Enable detrending and/or bandpass filtering by ticking the corresponding boxes.
      ii. Adjust the values in the edit boxes as needed (Figure 9).
      **Figure 9.**
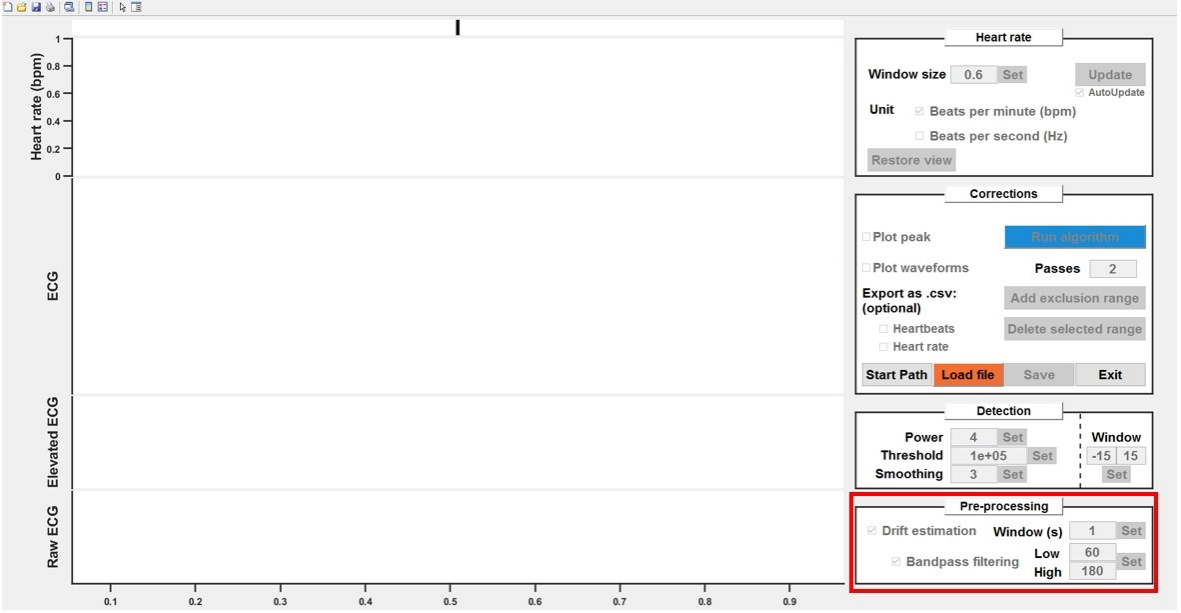
Pre-processing of raw electrocardiogram (ECG) signals. Detrending and bandpass filtering options can be enabled by ticking the respective boxes. Adjust the values as needed.
  3. Transformation of the ECG signal.
    The ECG signal is elevated to the power of n (where n is an even integer) to amplify heartbeats compared to the background. Typically, elevating the signal to the power of 4 yields optimal results, but it can be experimented with different values empirically. The resulting signal is then smoothed with a Gaussian kernel large enough to encompass one heartbeat, creating smooth peaks.
*Note: Some ECG signals may contain noise with similar amplitude and frequency (sharp artifacts). In such cases, reducing the smoothing window size can be beneficial, preventing noise from merging with the peaks. However, this may result in an increased number of detected peaks. In moderate cases, the algorithm can handle this effectively.*
    Adjust the values in the edit boxes and refine with the following steps if necessary.
  4. Thresholding and putative heartbeat extraction.
    The elevated/smoothed signal is thresholded to detect potential heartbeats. The "Window" parameter determines the number of samples before and after the threshold crossing to be selected as a single waveform (potential heartbeat).
*Note: This parameter is mainly important for the algorithm step but does not need to be extremely tight around one heartbeat. What matters is that it captures the whole beat without overlapping with another one.*
    - On the *ECG* plot, identify the window with the smallest amplitude for heartbeats and zoom in on that area. Adjust the blue line to cross the peak corresponding to the smallest heartbeat (approximately 1/5 of its height; Figure 10).
    - Check the heart rate curve for sharp, unnatural drops indicating missing heartbeats. Zoom in on suspicious areas to confirm the heartbeats were not detected (no filled diamond) and adjust the threshold if necessary.
*Note: It is crucial to make sure that all heartbeats are detected at this step. Further steps involve manual cleanup that could be wasted if some beats are missing, since readjusting the threshold will reset the rest.*
    - If this is one of the first recordings in these conditions, tick the *Plot waveforms* checkbox. This will display, for each thresholded peak, the corresponding waveform directly on the *ECG* trace. Adjust the values for the *Window* parameter (number of samples before and after threshold crossing) so that the main features of a single beat are included (as visible on the overlay).
*Note: Keeping the waveforms and/or peaks always plotted can lead to a decrease in GUI reactivity.*
      **Figure 10.**
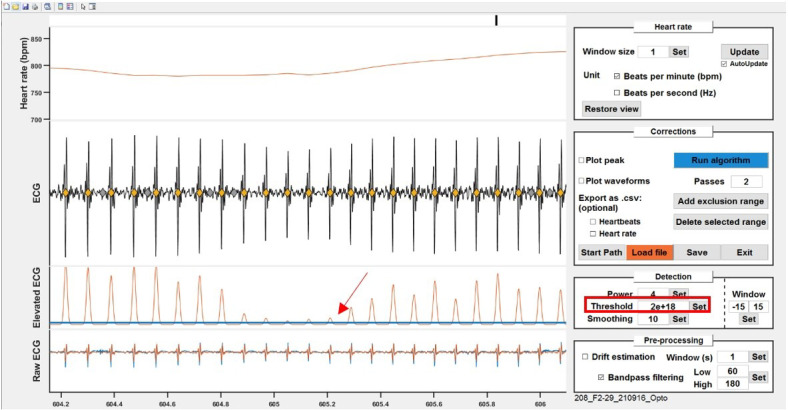
Thresholding to detect potential heartbeats. Adjust the threshold (blue line) either by dragging the line or by typing in a value to cross the smallest peak in the recording.
  5. Run the algorithm.
    The algorithm operates iteratively, systematically analyzing successive putative heartbeats. Here is a concise overview of its operation:
    Template Creation: The algorithm begins by constructing a representative waveform derived from all the putative heartbeats. This template serves as a reference for comparing with potential peak candidates, computing correlation values.
    Iterating: Starting from an area where at least a few consecutive peaks have a good correlation score, it will progress to the next peak and iteratively do so from peak to peak.
    For each peak, the algorithm computes a mean score from the peak’s correlation score and a *position* score. To obtain the position score, the algorithm creates a Gaussian probability curve based on previous (previously validated) beats. If the following peak *falls* close to the average from the previous intervals, the score is maximal and slowly decreases for values that deviate.
    When either or both scores have critically low values for all putative heart beats in a given time window, the algorithm engages in parallel paths. In each path, it selects one of the closely located peaks as the valid heartbeat and discards the others. For each path, the algorithm processes the corresponding ECG signals for a few seconds of recordings, as mentioned here. The algorithm then computes a global score for the segments obtained from each possibility, considering an aggregated score derived from correlation and position scores of all the peaks in the segment. Based on the global score, the algorithm decides which of the initial candidates to retain as valid heartbeat and keeps processing the signal further.
*Note: The algorithm is purposely tuned to clean sections where peaks can be safely identified as heartbeats and ignore the rest so that it can be processed manually.*
  6. Manual post-processing.
    The heart rate curve is inspected for suspicious patterns. In cases where the algorithm struggles to distinguish between noise and actual heartbeats, but the experimenter can confidently identify *true* heartbeats, it is safe to discard the noise peaks and retain the authentic heartbeats. However, if the experimenter has any doubt, we recommend excluding the time period starting from (and including) the last clearly identified *valid* heartbeat up to (and including) the first subsequent valid heartbeat.
    - Choose a zoom magnitude that allows to see anomalies in the heart rate curve (typically approximately 200 s). Browse the recording with the slider line on top or by pressing left/right arrows on the keyboard.
    - Check for anomalies (Figure 11).
      i. A typical example is when a noise peak is taken instead of a very close heartbeat; because the noise peak is *too early*, it creates a sharp and very transient rise in heart rate, and when the sliding window used for computing the heart rate goes past it, it then creates an equally sharp *dip*. This biphasic noise is probably the most common and easiest to identify.
      ii. Most of the time, when there are sections that the algorithm could not successfully clean, the manual processing consists in unclicking the *noise* peaks and/or setting the whole section to be excluded. Excluded ranges can then be set as NaN values for further processing. To exclude a range, click on *Add exclusion range* and draw a rectangle on the *ECG* plot. Overlapping ranges are automatically merged, and ranges can also be deleted by clicking on them and then clicking *Delete selected range*.
*Notes:*
*1). Except when dealing with occasional recordings of suboptimal quality (poor signal-to-noise, artifacts, etc.), manual post-processing is extremely fast. It takes only a few recordings to get familiarized with heart rate curve and how they typically look and efficiently spotting problematic ranges with a quick glance.*
*2). It is better to use a small window for heart rate processing at this step, as it does not smooth out the anomalies as a bigger window would. 0.6 s is usually enough to not create gaps in the heart rate and allows for good identification of problematic ranges.*
*3). When manually ticking/unticking several peaks at once, it is better to untick AutoUpdate while (de)selecting and then tick it back afterwards. This saves time by preventing the GUI from refreshing after each operation.*
*4). Excluded ranges are displayed as filled grey areas. The empty periods following then, and ending with a dashed line, represent the periods for which heart rate cannot be processed because of the excluded section, but are not, per se, excluded ranges.*
      **Critical:** It is highly advisable to pre-process each recording perfectly as it ensures that they can be used safely for any kind of subsequent analyses, without compromising the results by adding noise or biases to the quantifications.
      **Figure 11.**
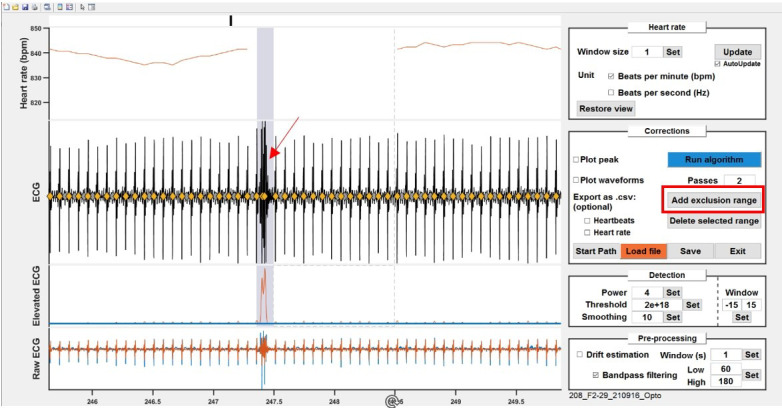
Check for anomalies. Example of a noisy recording snippet. Noise peaks are easily visible by sharp, transient rise and dips on the heart rate curve (top). Unselect the falsely picked peaks or exclude ranges by clicking *Add exclusion range*.
  7. Saving.
    The GUI exports two files:
    - A “_HeartBeats.mat” file, containing heartbeat timestamps and exclusion ranges.
    - An “_ECGLog.mat” file, containing the analysis parameters and information regarding the experimenters and the date.
*Note: An option can be enabled within the function to export the heartbeats and exclusion ranges as a .csv file.*
- **Obtaining the readout from the raw heartbeats**
  The same method as the one used by the GUI can be implemented to derive heart rate from heartbeats.
  1. Choose a fixed window size, like 0.6 s.
  2. For each heartbeat, count the number of heartbeats that occurred in that 0.6 s window before the current beat.
  3. Divide this count by the time difference between the first beat in the window and the current beat.
  4. Parse the exclusion ranges and set all the values that fall in the corresponding time windows (plus the size of the averaging window) to NaN. This allows to average between mice/periods without having artifacts from the *bad* ranges.
  5. (Optional) This method offers a quick and efficient way to derive heart rate from heartbeats. However, it results in irregularly spaced data points because there is one data point for each heartbeat. To make it suitable for averages or when creating peristimulus time histograms, it is essential to resample the data to a fixed sampling rate so that the data is evenly spaced.
*Note: When using this method to process heart rate from heartbeats, it is important to understand that the window ends precisely at each beat. This means that the heart rate curve shows immediate increases in heart rate with each beat. However, it is crucial to recognize that the decrease in heart rate is more gradual. Different alignment options could be chosen, such as centering the window on each beat or starting it at each beat, among others, depending on your technical preferences. Regardless of the chosen alignment, it is essential to keep this in mind when interpreting the data, as it can affect the perception of heart rate changes.*
*Use the GetHeartRate.m function to replicate this step.*
- **Summary of analyses/quantifications that can be performed**
  1. Global averages.
    Why: To globally compare a particular readout between general conditions (e.g., species, context).
    How: Perform a long recording, pre-process the data, and obtain one average per individual.
    Statistics: Depends on the characteristics of what is compared (Figure 12).
    - If the values come from a single group from measures repeated at different time points (for instance during the development of a pathology) or under different conditions (sated vs. fed): repeated measures one-way test + post-hoc tests.
    - If the values come from a single group but different cohorts (for instance different drugs are tested): a one-way test + post-hoc tests.
    - If the values come from several groups (for instance wild-type vs. mice with specific knocked-out genes): a one-way test + post-hoc tests.
    - If the values come from several groups recorded at different time points (for instance comparing the development of a model at different weeks between mice that received the treatment vs. control mice): a repeated measures two-way test + post-hoc tests.
    - If the values come from several groups receiving different treatments, with one treatment per cohort: a two-way test + post-hoc tests.
    Interpretations: Depends on the test.
*Note: For all these cases, one important aspect is to properly inform the statistical software about the matched conditions. The exact choice of the test depends on the number of groups and whether certain conditions are fulfilled (e.g., normality, homoscedasticity), which falls beyond the scope of the current protocol. Same goes for the interpretation: considering main effects/interactions when available and when to interpret them, and post-hoc comparisons.*
    **Figure 12.**
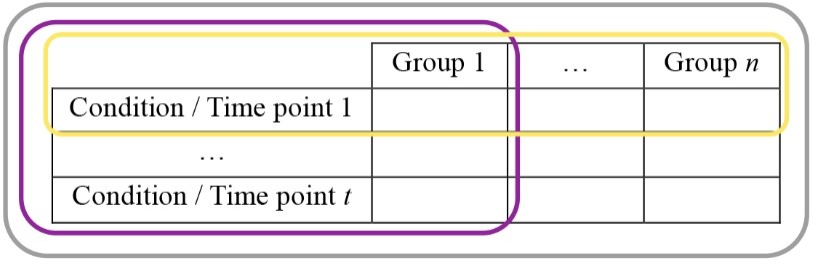
Typical data holder table. (i) n groups could be recorded under t conditions or time points each (the whole table, grey contour), allowing the use of two-way tests. (ii) In case n groups were recorded for a single condition (yellow contour), or (iii) if a single group was recorded under t conditions, a one-way test would be required. Both cases (i) and (iii) would require taking into account *repetitions* if the same individuals were undergoing the different conditions.
  2. Average whole traces.
    Why: To observe the evolution of a signal during a recording, potentially between two groups/conditions.
    How: Perform a long recording, pre-process the data, and obtain average curve(s) from matching conditions.
    Statistics: If the statistical analyses are to match the curves (which could still only be showed as example/descriptive data), time needs to be one of the dimensions, which de facto changes all statistics to repeated measures tests. Post-hoc tests could then inform about specific time periods that are significantly different (within groups or across). Same principles as above apply—but this time with potentially more factors, which can lead to three-way analyses and/or sub-selecting the data by conditions and operating statistical tests on meaningful subsets of factors. Alternatively, group-matched fitting and associated statistical comparison could be used.
  3. Peri-stimulus time histograms (PSTH).
    Why: To observe changes happening around a synchronizing event (stimulus, behavior, etc.), potentially between two groups/conditions.
    How: Perform a recording, pre-process the data by aligning it around the events, and obtain average curve(s) from matching conditions.
*Note: Data can be normalized, typically to a baseline, by subtracting the mean and, optionally, dividing by the standard deviation (resulting in a z-score from the baseline). However, it is crucial to consider whether the baseline itself exhibits intrinsic differences, as this could either obscure or introduce an artificial effect during the normalized period (see section D for more details and examples).*
    Quantifications/statistics:
    - One way is to treat PSTHs like the average whole traces and perform RM analyses.
    - Another is to compare before/after periods directly with paired tests.
    - Finally, more tailored analyses could be used when they make sense. For example, a single peak or trough value can be extracted in a specific time window and group comparisons can be conducted on these individual per-mouse values.
*Note: When constructing PSTH, particularly when presenting data dispersion on the curve (using either standard error or standard error of the mean) and choosing/reporting statistics, the precise method of averaging and counting is decisive and should be transparently documented.*
*Pooling all events from all animals is justifiable in specific cases but inherently reduces the apparent data dispersion while increasing statistical significance. Furthermore, the number of events per mouse can vary based on experimental designs or quantification methods, especially when the synchronizing event involves behavior. Consequently, relying solely on a global average can skew the results towards specific mice within the study. It is advisable to obtain an average per experimental unit (one recording and/or one animal) to use as a value for the PSTH and statistics. The range of total number of events could still be mentioned where appropriate as average number ± deviation.*
- **Interpreting the data**
  Direct interpretation of the analyses presented above should not pose any significant challenge beyond typical statistical results interpretation. However, the method of choice and data sub-selection require careful consideration.
  For studies based on event-induced changes, it is tempting to focus exclusively on PSTH analyses; conversely, for studies comparing groups, it seems straightforward to compare average heart rate from group A with average heart rate from group B, and so on. However, before performing any quantification or further analysis, it is often useful to take a global look at heart rate curves. To illustrate the importance and benefits of such approach, we provide below a few examples of key principles and potential confounds or insights that might be overlooked during a *blind* analysis (examples in Figure 13, summarized in Table 1) before discussing their underpinnings and then generalizing and giving general directions on how to explore new datasets.
*Note: These examples primarily revolve around heart rate changes associated with behavioral immobility, as it is the central focus of the study that generated the data set from which they were selected. Nonetheless, the underlying principles and key takeaways should be relevant and applicable to a wide range of conditions.*
  **Figure 13.**
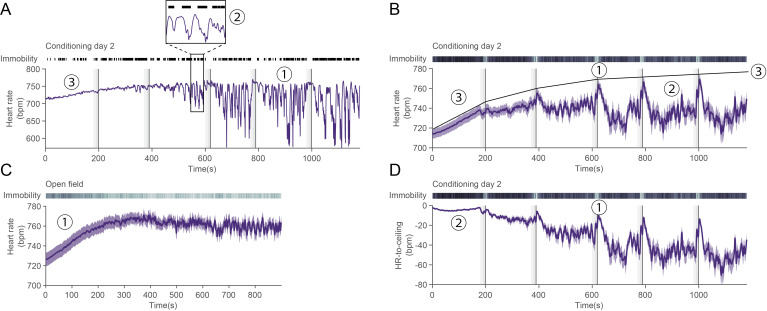
State-dependency of heart rate values. A. Representative heart rate trace for a single mouse over the course of a second fear-conditioning day. Black lines on top depict immobility bouts, vertical dashed lines represent the compound conditioned stimulus (CS), and vertical dark lines the US presentations. (1) At first, it could be interpreted that heart rate variability (HRV) is increasing over time, but (2) heart rate fluctuations are tightly correlated to immobility behavior. (3) There is a slow rise in heart rate at the beginning of the recording that could be attributed to context-re-exposure and therefore *fear*. Additionally, while similar values can be reached during e.g., (1) as during the early phase (3), they are unlikely to have the same mechanistic underpinnings. B. Average heart rate for n = 33 mice during conditioning day 2. (1) It looks as though there is a peak only during CS phase, and (2) baseline-like levels in between. While this undeniably captures an average phenomenon, it does not reflect single mouse curves (e.g., in A.), in which there seems to be a toggle between bounded high and low values. (3) Such theoretical maximum (*ceiling*) is represented onto the average curve. C. Average heart rate curve for n = 25 mice in an open-field recording. (1) A similar early increase as in conditioning day 2 [B. (3)] can be seen. This suggests that such increase is unrelated to conditioning itself. D. *Detrending* heart rate based on the theoretical maximum in B (3) allows for a more robust and faithful interpretation: (1) heart rate decreases between CSs and peaks during the second half of the CS and the shock. (2) This also leads the early rise to be *normalized*, which prevents wrongful interpretation about absolute heart rate values. It also homogenizes the values between mice as visible by the very low variability when compared to B (3).
  **Table 1.** Examples of potential observations of heart rate characteristics and their direct and adjusted interpretations

Observation	Direct interpretation	Closer look	Adjusted interpretation
A(1) HRV is high	Fear conditioning increases HRV	A(2) *HRV* here actually reflects immobility bouts that are correlated with HR decrease	Fear conditioning conditions increase immobility bout probability
A(1) HRV is increasing over time	Fear conditioning increases HRV over time	The amplitude of immobility-related decreases in heart rate increases over the course of the recordings	Some latent process over the course of the recordings globally affects the amplitude of heart rate changes (*Rigidity*). This is not specific to threat exposure, but threat exposure changes the magnitude of this phenomenon
A(3), B(3) HR increases at the beginning of the recording	Re-exposure to the conditioning context increases HR	C(1) HR increases at the beginning of the recordings in other contexts	Something in the recording procedure leads to an early increase in HR
B(1) HR increases during CS/US pairings, but B(2) remains at a constant level in between	CS/US increases HR	D(1), A(1) HR decreases during immobility bouts that are frequent outside the CS/US pairings, and increase during the second half of the CS/the US	HR changes reflect immobility probability changes as well as * rigidity* changes
  These few points highlight several key principles:
  1. State dependency
    It is common, particularly in human studies, to discuss heart rate in terms of a constant value and compare general metrics across individuals with diverse ages, fitness levels, and health statuses. However, it is evident that heart rate is a highly dynamic parameter, influenced by a spectrum of biological processes and environmental elements, operating on different timescales. Circadian rhythms modulate HR, HR increases to match the body’s needs when exercising, and most importantly, emotions drastically affect HR. Hence, when considering these average values, it is often implied that they correspond to the *resting heart rate* obtained under equivalent and somewhat controlled circumstances. Similarly, in mouse studies, a single recording should yield a valid per-individual average as long as the conditions remain consistent between the values being compared. Nevertheless, for mice, interpretations can quickly become delicate, with the slightest variations in the conditions. While avoiding excessive determinism, the aim is to work within a framework that acknowledges the accessible factors, enabling a balanced interpretation and robust conclusions.
    - Behavior
      Behavior is a critical determinant of heart rate values in mice. For instance, entering or leaving a *defensive* immobility bout, commonly termed *freezing*, leads to rapid and drastic changes in heart rate. Rearing causes a transient increase in heart rate, and many other behaviors are likely associated with heart rate fluctuations. Interestingly, the increase in heart rate associated with locomotion is often masked in many typical recording conditions (see Section B). In general, the most pronounced heart rate changes can be traced back to a behavioral change.
      Generally, the most significant short-term heart rate changes can be attributed to behavioral shifts. Even within the same behavior, the cardiac response can depend on intrinsic properties, such as the duration of the behavioral bout (for example, immobility bout duration is positively correlated with the heart rate decrease amplitude).
      • When averaging over extended periods, the resulting average heart rate may heavily depend on the proportion of certain behaviors (e.g., if the mouse displays many immobility bouts, the average will be low) and their respective characteristics, which can be influenced by different factors.
      • Analytical power and meaningfulness of the results can be enhanced by comparing heart rate values during similar behaviors and even accounting for specific behavioral bout characteristics.
      → Investigate if individual behaviors are associated with consistent, time-locked heart rate changes (i.e., one behavioral bout leads to a stereotypical heart rate change).
      → Explore if there are time periods enriched in certain behaviors that exhibit overall higher or lower heart rates.
      → Incorporate these factors into the analyses by matching behaviors and periods accordingly.
    - Context
      The diffuse *threat level* of the context, and consequently, the anxiety it generates, affects average heart rate values (even for matched behaviors; Signoret-Genest et al., 2023). This is true both for a paradigm as a whole (e.g., open field vs. small arena) as well as within specific areas of certain paradigms (e.g., closed arms vs. open arms in an elevated plus maze). This perception of diffuse threat is shaped by inherent species-specific evolutionary responses (e.g., open spaces being associated with an increased risk of predation) and may be influenced by other factors such as prior history or individual traits.
      Surprisingly, the level of contextual threat also influences the magnitude of heart rate changes associated with the same behavior: bradycardia associated with immobility is reduced in higher-threat environments.
      Notably, the baseline threat level/stimulus induced by standard recording procedures can be sufficient to obscure any increase in heart rate associated with locomotion, as the baseline is already elevated (Andreev-Andrievskiy et al., 2014).
      In extreme cases, presenting the same stimulus in different contexts can elicit opposing heart rate responses (see also e.g., Signoret-Genest et al., 2023).
      • If two different recordings are performed to compare two conditions (e.g., two drugs) but in two areas that are too different, it could reflect an influence of the area instead of that of the treatment.
      • When averaging across a session recorded in a paradigm with areas associated with different threat levels, the time spent in each area will influence the resulting average.
      • Again, accounting for the potential impact of context can increase statistical power by controlling for the variability it otherwise induces, which also constitutes a meaningful result in itself.
      → Take these considerations into account for the analyses (ensure context/areas are matched, assess context/areas' independent influence).
    - Heart rate modulation at different time scales
      Changes in heart rate related to behavior operate on the timescale of a behavioral episode—ranging from fractions of a second to a few dozen seconds. Contextual effects may extend over an entire recording or specific periods within a recording when subareas present differences. Additionally, slower processes can manifest, as demonstrated in the provided example (an increase in heart rate due to the recording procedure and a gradual augmentation in the amplitude of cardiac responses).
      → Again, it is advisable to account for these factors in the analyses, seeking out patterns and correlations across different scales. Decomposing the signal in such a way may allow for the mitigation of these factors’ influence on the data as well as their comprehensive characterization.
    - History/individual characteristics
      Exposure of mice to fear conditioning, for example, is likely to lead to subsequent heightened levels of anxiety. Beyond the observable behavioral shifts and associated direct heart rate changes, one would anticipate consequential alterations in heart rate patterns. Metabolic challenges at earlier time points may also have a lasting impact on cardiac responses, and various other biologically significant states can reasonably be expected to induce or modulate changes in heart rate (e.g., satiated vs. fed states). Taking these factors into account in specific research areas is crucial as they have the potential to significantly influence outcomes.
      → In some cases, conducting longitudinal analyses and recognizing the individuality of mice entering a session (beyond the obvious treatment differences) can provide valuable insights by introducing a level of complexity that helps elucidate variability.
    - Preexisting state
      *Baselines* are also a non-negligible source of variability, often hidden in the fine methods details. Leaving mice to habituate before starting the recording/procedure or not can lead to major differences—up to the point of inverting the heart rate responses. But even at the level of smaller scale events, such as an individual behavioral bout or the presentation of a stimulus, the pre-state of the mouse is a critical determinant of the following cardiac changes. In some cases, the relevant toggle for heart rate is entering or exiting a specific behavior, in which case transitioning from or to another one, respectively, does not matter.
      → It is sometimes worth thinking of transition from one state to another rather than simply entering/exiting one state (e.g., a behavior).
      → A corollary to this is that normalization of the signal to baseline is a double-edged sword since the baseline might not be homogenous between episodes and/or conditions and present intrinsic meanings on its own. For example, if a behavior is correlated to a decrease in heart rate globally, but one episode occurs during a state where the heart rate is already low, it might not decrease any further. Normalizing by the baseline would look as though there is no change, when the most relevant information is that the heart rate is (still) low.
    - Interaction
      Different aspects could interact with each other, yielding potentially paradoxical results if not disentangled. For instance, if a certain heart rate change occurs at a very specific time point, and if mice happen to be probabilistically in a specific area around the same period, the area-effect could modulate the otherwise non-causally related heart rate change.
      → As a last step, the different elements should be pieced together.
  2. Umbrella terms and semantics
    Trying to fit an observation “into a box” too early on can be problematic, as it leads to skewed data exploration and interpretations that are biased by higher level concepts and semantics and taken away from the low-level data description.
    A prime example is the HRV, a widely used metric for studying human heart rate, making it a potentially valuable tool for mouse studies with translation in mind. However, it is worth noting that HRV encompasses a wide range of analyses. While descriptive and comparative results can be useful for diagnostics and revealing hidden states, the field faces a challenge in terms of lacking a solid mechanistic grounding and understanding. This can make biological interpretations somewhat precarious, especially as the increasing popularity of HRV can sometimes lead to overreaching interpretations that are not grounded in facts.
    Most HRV analyses rely on long recordings, for which they produce a single value per readout. So-called time domain analyses look directly into beats intervals. The Root Mean Sum of Squared Successive Differences (RMSSD) is one of the most commonly used (Shaffer et al., 2014). However, because these are so closely related to beats intervals, they are inherently strongly correlated to changes in heart rate at a small-time scale. For that reason, even larger time scales, particularly in mice, should be controlled for the points mentioned in step D1.
    Frequency-based analyses of HRV look at the representation of certain frequency bands in the heart rate signal (that is, how much it oscillates at certain rhythms). While they provide sometimes useful biomarkers, their biological underpinnings are unclear and there is no clear consensus. We recently applied frequency analyses to our signals to quantify a macroscopic, visible oscillation: the equivalent of the Mayer-Waves in humans, which are the oscillations hypothesized to originate from baroreflex loops. Because their amplitude was affected in a similar manner as some of the other, simpler, heart rate changes (increases/decreases), it provided us with a bidirectional validation of our hypothesis—that a latent mechanism related to a baroreflex curve tuning was constraining heart rate changes.
    A last type of HRV analyses exists: the non-linear analyses, which suggest that heart rate is inherently *fractal* (it repeats patterns at different scales). While showing promises for diagnosis, it suffers from a lack of biological interpretability.
    → We would advise to use HRV analyses either with translatability to humans in mind, or to characterize and quantify a very obvious oscillatory pattern in heart rate that cannot be explained by other means, as many changes could otherwise be inadvertently confused for HRV.

## Validation of protocol

This protocol or parts of it has been used and validated in the following research article:

Signoret-Genest et al. (2023). Integrated cardio-behavioral responses to threat define defensive states. Nature Neuroscience (all figures).

We are providing a small data set along with the current protocol, allowing to access examples of raw ECG data as well as the corresponding pre-processing and analysis procedures. Additionally, we provide open-access code that can be applied to any steps for testing and experimentation.

## General notes and troubleshooting

**General notes**

1. We chose to use 6-pin Omnetics connectors because of their minimal size and weight, which allows to combine ECG recordings with techniques requiring additional elements to be fixed onto the skull, and because we might occasionally require additional channels. These connectors are of high-end quality and provide perfect connections but can increase the per-mouse costs, even though they are reusable with proper care (see section C). Simpler three-pin connectors might be used instead; the general procedure and important tips remain the same.
2. Manufacturing the patch cable may initially pose challenges due to the fragility of the wire. In terms of selecting the cable model, the chosen reference is relatively costly but offers two significant advantages: a) it is exceptionally thin and lightweight, and b) it is shielded, making it suitable for recording in conditions where noise cannot be avoided (or simply dealing with usual ambient noise). On the other hand, this comes at the cost of being pretty delicate. Considering these points, the decision could be made to switch for a cheaper/sturdier reference if it seems more beneficial.
3. The ECG implantation described here is the least invasive possible in terms of number of implanted electrodes and providing the most stable recordings over several weeks. It allows for accurate and robust heartbeat detection and therefore reliable heart rate extraction. However, with the setup and parts proposed here, it can effortlessly be expanded to four channels for applications that might need more in-depth analyses of the heartbeats themselves (waves). Since the cable is already made so that it can feed the preamplifier signals from more than two channels, it only requires to solder two more wires to the connectors and implant the four resulting electrodes at the desired place on the mouse. It is then probably best to record only the direct output from each channel and take care of the specific ECG derivations offline.

**Troubleshooting**

**Problem 1: The ECG signal is not visible**

Possible cause #1: There is too much noise to see the ECG.

Solution #1: See Problem 2.

Possible cause #2: Something is wrong with the amplifier parameters or the cabling, etc.

Solution #2: See section D from Procedure.

Possible cause #3: Something is wrong with the implantation.

Solution #3: Assess the surgery quality, if needed post-mortem, to understand what could have gone wrong.

Possible cause #4: Something is wrong with the connector preparation or the cable integrity.

Solution #4: Check the cable for shortcuts or lack of connection and check any new connector before implanting (making sure the soldering is of good quality and that each pin connects to the end of its corresponding wire).

**Problem 2: The signal is noisy**

Possible cause #1: The signal and recording system are fine but there is no ECG, so the baseline looks as though we have only noise.

Solution #1: Troubleshoot for no ECG (implantation issue, cable/connector issue).

Possible cause #2: Something is wrong with the amplifier parameters.

Solution #2: See section D from Procedure.

Possible cause #3: The system is picking noise, in particular the ambient “hum” (noise coming from AC-powered equipment). The shielded cable decreases the impact of that common issue with electrophysiological recordings but might not prevent it entirely. This is very likely to happen for a new setup. Check for 50 Hz (or 60 Hz depending on the power-grid characteristics) and harmonics in the spectrogram of a recording.

Solution #3: The system will need to be *grounded* in such a way that the noise is gone or at least decreased. There are general principles as to how to ground a system but no unique solution. In case nothing works, it might be best to contact the support from the amplifier company (NPI in the case of this setup).

**Problem 3: The signal looks fine sometimes, but some sharp artifacts render the rest of the recordings unusable**

First, assess whether it occurs for a single individual or all of them.

*If it happens for a single individual:*

Possible cause #1: One or several wires are not properly fixed (anymore). This is likely to be due to a problem during the surgery. The wires could have been slightly too short, leading to some tension, ultimately leading them to move away, or the stitches on the muscles were suboptimal, or the ball of glue at the tip was not big enough/stable enough, or a mix of several factors.

Solution #1: Once the technique is implemented and the experimenter is experienced enough, these particular issues should become rare. In most cases, it seems better to address the problematic elements upstream (e.g., having wires long enough, properly stitched), than to resort to a second surgery to try and fix things, which might not even be possible.

Possible cause #2: Similar to cause #1, but a single wire is affected.

Solution #2: Change the settings to disable differential recordings and check each wire’s individual signal. If the environment is relatively *noise-free*, differential recording is not mandatory and it could be that the differential signal looks bad because one wire has an issue, while the single channel’s signal from the other would look fine.

Possible cause #3: The electrode picks a lot of muscular signals (electromyogram, EMG), which typically looks like bursts on the recording. They are present to varying degree in some animals, but it becomes problematic when their amplitude is greater than that of the ECG.

Solution #3: That particular animal was implanted at a suboptimal location and/or the electrode was stripped too much or not enough; it cannot be solved but can serve as feedback for later implantations.

*If it happens for all individuals:*

Possible cause #1: The head-to-headstage cable is touching something.

Solution #1: Make sure the cable is hanging from the middle of the arena as much as possible and not touching anything else than the thread holding it up via the pulley.

Possible cause #2: The cable is damaged. The cable used is ideal because it is shielded and light weight, but it is also fragile. A moment of inattention leading to a mouse grabbing and biting the cable can lead to some bite marks that can produce various effects depending on what is damaged:

- Breaching the shielding can let some environmental noise through.

- Breaching the individual wires’ insulation can lead them to sometimes contact other wires or the shielding sheath, creating artifacts as motion is applied to the cable.

- If a single wire is partially severed, it could intermittently *disconnect*.

Note that bending the cable too hard or repeatedly on the same place can also lead to damage that is not visible, with similar consequences.

Solution #2: Check for bite marks along the cable and assess how bad the damage is. In case of no external damage, carefully palpate the cable on all its length to locate potential breaks inside the insulation.

If there is no time to make another cable (an experiment is running and cannot be postponed), using tape to tightly wrap the cable can hold the wires in place for some time, which provides a temporary solution. Regardless, the cable needs to be changed; it is wise to always have a second cable ready just in case.

And as a more general fix: the setup could maybe be improved so that the cable cannot be easily grabbed (enough tension to pull it when needed and keeping it from hanging too low) and making sure the experimenter keeps a close eye on what is happening, so that he can intervene whenever required.

Possible cause #3: There are some rather slow and somewhat cyclic drifts at play, but the filtering is set in such a way that it looks like artifacts during some turning points.

Solution #3: Check Problem 3.

**Figure 14.**
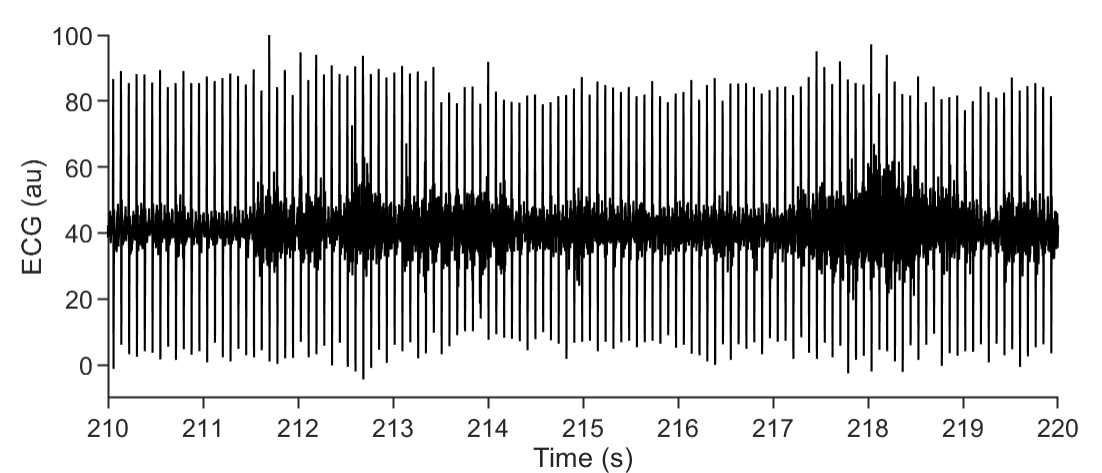
Electrocardiogram (ECG) contamination by electromyogram (EMG) signal. Representative raw ECG trace (only filtered online by the amplifier), showing heartbeat signal and EMG contamination (looking like thicker baseline noise, e.g., prominent at t = 218 s). In this case, the amplitude is smaller than the ECG signal, but in some recordings the EMG signal could prevent ECG analysis.

Possible cause #4: The electrode picks a lot of muscular signals (electromyogram, EMG), which typically looks like bursts on the recording (Figure 14). If this happens more or less systematically and not anecdotally and is not restricted to bursts of very small amplitude (in theory, if the amplitude is smaller than the heartbeat’s signal, it should be workable but still denotes a systematic issue), two things could be checked:

Solution #4a: The implantation sites are not optimal and must be readjusted.

Solution #4b: The electrodes’ material might not have ideal properties. Try to use the reference provided here, or at least, change from what is currently used.

**Problem 4: The signal looks fine sometimes, but some slow oscillations are superimposed**

Possible cause: The recordings are combined with another device and the shielding is either damaged or insufficient.

We encountered such issues in two different cases, which can help understand others:

The ECG cable is bundled with a data transfer cable for a different type of recordings.

In our case, we bundled the ECG cable with the cable for a miniaturized head-mounted microscope (*miniscope*). That microscope relies on a wet-lens to achieve focus on the region of interest and can quickly shift between several depths of focus. This requires *reshaping* the wet lens with different currents that cause specific offset when the focus is held.

Solution #1: We implemented the current shielded cable, which works fine into preventing all noise contamination. If the contamination comes back, that is a sign that the shielding is damaged.

The animal is placed in an electromagnetic field.

For some other experiments, we needed to deliver electromagnetic stimulations. The stimulations were ranging between 10 and 50Hz, 50% duty cycle. At each pulse, a biphasic deflection could be visible on the ECG (a rise then a decrease).

Solution #1: The shielded cable was not enough to prevent noise contamination, as the entire subject was bombarded with it. We were however able to record the current pulses driving the stimulation to retrieve their timing. This allowed us to determine and fit a model for the noise by looking at the signal at each onset/offset of the stimulation. Fitting the model to each stimulation then allowed to subtract the estimated noise and retrieve an almost *clean* ECG signal that could then be pre-processed as usual. This can in theory be extended to different types of noise contamination that follow a discernable pattern. Alternatively, under certain conditions, one could implant the reference wire closer to the other electrodes, so that the noise would be more similar and therefore just subtracted.

**Problem 5: The head cap falls off**

Possible cause #1: The surface of the cap on the skull is not enough.

Solution #1: Remove a bigger patch of skin to build a larger head cap.

Possible cause #2: The glue was applied on a wet skull.

Solution #2: Dry the bone before adding the glue. Some minor bleeding can sometimes occur from the bone itself; do not use bone wax as it would decrease glue’s adhesion but gently scrap the bleeding area with a blunt tool instead (consider using an appropriate rugine).

Possible cause #3: The surface of the skull was not scratched enough.

Solution #3: On the dry bone, do not hesitate to draw a very clear grid pattern with the back of a scalpel blade. This is critical for the head cap to be firmly fixed.
